# Investigation and influencing factors about well-being level of elderly chronic patients during COVID-19 postpandemic period in Beijing

**DOI:** 10.1097/MD.0000000000028976

**Published:** 2022-03-04

**Authors:** Chen Wu, Yu-Xuan Liu, Tie-Jun Liu, Xu-Ling Yan, Yu-Xi Zhao, Hong Zeng, Tian Zhou, Ping Rao, Lan-Ying Sun, Yang Jiao, Jia-Ning Xi

**Affiliations:** a Beijing Rehabilitation Hospital of Capital Medical University, Beijing, China; b Beijing Municipal Health Commission, Beijing, China; c Peking University Third Hospital.

**Keywords:** coronavirus disease 2019, postpandemic period, elderly patients with chronic diseases, influencing factors, well-being level

## Abstract

The Corona Virus Disease 2019 (COVID-19) pandemic has huge impacts on the world, including human health and economic decline. The COVID-19 has severe infectivity, especially the elderly with chronic diseases will cause various complications after infection and accelerate the disease process. In addition, COVID-19 will also affect their mental health. Therefore, the mental health of elderly patients with chronic diseases cannot be ignored. The aim of this study was to investigate the well-being level of elderly people with chronic disease during COVID-19 postpandemic period in Beijing and analysis related influencing factors, so as to provide a basis for improving the well-being level of elderly chronic patients during the postpandemic period.

Elderly patients with chronic diseases who met the inclusion criteria in 5 different administrative regions in Beijing were selected to carry out a questionnaire survey. The contents of the questionnaire included general data, the Memorial University of Newfoundland Happiness scale and the awareness situation of the COVID-19 pandemic. A total of 500 questionnaires were distributed by WeChat and 486 valid questionnaires were collected. The *t* test and one-way analysis of variance were used to compare Memorial University of Newfoundland Happiness scores between 2 or more groups, multiple linear regression analysis was used to conduct multiple factor analysis to explore the related factors about well-being level of elderly chronic patients.

A total of 109 cases (22.43%) were evaluated high well-being level, 319 cases (65.64%) were evaluated moderate well-being level and 58 cases (11.93%) were evaluated low well-being according to the Memorial University of Newfoundland Happiness (MUNSH) scores rating. The multiple linear regression indicated that the education level, number of chronic diseases, medical expenses, frequency of children's visits, taking care of grandchildren or not, and group activity frequency significantly affected the well-being of patients with chronic diseases during COVID-19 postpandemic period in Beijing (*P* < .05).

Most elderly patients with chronic diseases had moderate or above sense of well-being during postpandemic period, but we should still pay attention to the mental health of those elderly chronic patients with low education level, much comorbidity, more medical expenses, less visits by children, not take care of grandchildren and never participate in group activities.

## 1 Introduction

Since the outbreak of novel coronavirus pneumonia in 2019 (hereinafter referred to as “COVID-19 pandemic”), it has seriously affected the physical and mental health of residents and social economy and has become a global public health event.^[1,2]^ At present, the prevention and control of the pandemic in China has shifted from “emergency situation” to “normal postpandemic period” and people are gradually returning to normal life.^[3]^ Since COVID-19 has the characteristics of strong infectivity, high morbidity, rapid disease progression and general susceptibility of the population,^[4]^ especially the elderly patients with chronic diseases have more higher risk of COVID-19 infection, along with the risk of severe case and mortality also increased.^[5]^ In addition, China has a large base of senior citizens, and 76.3% senior citizens have at least 1 chronic disease.^[6]^ Therefore, it is vital to pay attention to the mental health of elderly patients with chronic diseases during COVID-19 postpandemic period.

## 2 Materials and methods

### 2.1 General information

The study was approved by the ethics committee of Beijing Rehabilitation Hospital of the Capital Medical University and all of the participants had signed an online informed consent.

Inclusion criteria:

1. Age ≥60 years old.
2. People with more than 1 diagnosed chronic disease including hypertension, diabetes, coronary heart disease, Chronic obstructive pulmonary disease (COPD), etc.
3. Have resided locally for at least 1 year.
4. No major mental diseases or dementia.
5. Can understand the questionnaire items or cooperate with respondents to complete the questionnaire.

Exclusion criteria:

1. Serious depression and suicidal tendency.
2. Cognitive impairment.
3. Mania or schizophrenia.
4. Malignant tumor patients.
5. Incomplete clinical data.

### 2.2 Research methods

#### 2.2.1 Survey tools

The questionnaire included the basic demographic information of research object, the Memorial University of Newfoundland Happiness (MUNSH) scale of Chinese revised version and relevant cognition of the COVID-19 pandemic. The basic demographic information consists of age, gender, occupation before retirement, number of chronic diseases and medical time, medical expenses, personal monthly income, frequency of children's visits, Take care of grandchildren or not, the number of hobbies. The investigation of the relevant cognition of the COVID-19 pandemic for instance medical condition, medical approach, support level for pandemic prevention and control.

#### 2.2.2 Well-being scale

The MUNSH scale of Chinese revised version^[7]^ was used to evaluate the subjective well-being. The MUNSH scale consists of positive affective (PA), negative affective (NA), positive (PE), and negative (NE) experiences with 24 items. The MUNSH scale = PA − NA + PE − NE + 24, with the score range of 0∼48 points. It can be divided into high level (≥36 points), medium level (12 < MUNSH < 36 points) and low level (≤12 points) according to the MUNSH score.

#### 2.2.3 Survey methods and quality control

From October 24 to November 12, 2020, a total of 500 questionnaires prepared by “Questionnaire Star” were distributed by WeChat according to the above inclusion and exclusion criteria, and a total of 486 valid questionnaires were collected. The effective recovery rate was 97.2%.

In order to ensure the comprehensiveness of the survey data, 500 questionnaires were randomly pushed by WeChat to 5 districts of Beijing. The questionnaires were filled anonymously, and the accounts that investigated would not be distributed again. The relevant terms could be explained in a unified statement. Questionnaire survey results can be directly derived from the survey program database to avoid the error of manual entry.

### 2.3 Statistics analysis

SPSS 25.0 software version 22.0 was used for data processing. The measurement data was expressed as mean ± SD, and the enumeration data was presented as a percentage. We assessed the discrepancy about MUNSH scores between 2 or more groups adopting *t* test and one-way classification of analysis of variance, while the multiple factors were analyzed by multivariate linear regression analysis. The *P* *<* .05 was considered as statistic difference.

## 3 Results

### 3.1 The MUNSH score of elderly patients with chronic diseases

The average MUNSH score of 486 elderly patients with chronic diseases in this survey were 28.35 ± 9.26 points, and the PA and NA score were 7.13 ± 2.82 points and 5.56 ± 1.79 points respectively, the PE and NE score were 8.06 ± 2.73 points and 5.28 ± 1.86 points. It can be seen that the PA and PE scores were higher than NA and NE scores (*P* < .05). A total of 109 cases (22.43%) were evaluated high well-being level, 319 cases (65.64%) were evaluated moderate well-being level and 58 cases (11.93%) were evaluated low well-being according to the MHSE scores rating.

### 3.2 Univariate analysis of MUNSH scores of elderly patients with chronic diseases in Beijing

Univariate analysis showed that the MUNSH scores of elderly patients with chronic diseases there were statistically significant differences in age, education level, number of chronic diseases, medication duration, medical expenses, frequency of children's visits, taking care of grandchildren or not, number of hobbies, and frequency of group activities (*P* < .05), as shown in Table 1.

**Table 1:** Comparison of subjective well-being scores among chronic elderly people with different characteristics in Beijing (N = 486).

Category	Cases/(%)	MUNSH scores ( $\bar{x}$ ± *s*)	*F/t*-value	*P*-value
Age (yr)				
60 ≤ 70	171 (35.19)	27.21 ± 10.36	3.185	.042
70 ≤ 80	164 (33.74)	28.13 ± 10.08		
≥80	151 (31.07)	29.98 ± 9.32		
Gender				
Male	238 (48.97)	27.96 ± 10.63	−0.848	.397
Female	248 (51.03)	28.72 ± 9.09		
Education level				
Primary school or below	143 (29.42)	27.12 ± 9.37	3.957	.020
Junior and senior high school	208 (42.80)	28.03 ± 9.21		
University or college education	135 (27.78)	30.15 ± 9.13		
Marital status				
Have spouse	218 (44.86)	29.14 ± 10.07	1.470	.143
Bereft of spouse	268 (55.14)	27.71 ± 11.13		
Preretirement occupation				
Peasant-worker	86 (17.70)	27.79 ± 10.83	0.520	.669
Worker	145 (29.84)	28.04 ± 9.18		
Leader	178 (36.63)	28.34 ± 9.74		
Veterans	77 (15.84)	29.58 ± 11.32		
Number of chronic diseases				
1∼2	135 (27.78)	31.32 ± 10.49	8.356	.000
3∼4	264 (54.32)	27.59 ± 10.65		
>5	87 (17.90)	26.05 ± 9.37		
Medical time (yr)				
<1	56 (11.52)	29.46 ± 9.38	6.141	.002
1∼4	186 (38.27)	30.07 ± 10.15		
>4	244 (50.21)	26.78 ± 9.96		
Medical expenses (RMB/mo)				
<300	134 (27.57)	30.13 ± 11.89	4.406	.013
300∼1500	186 (38.27)	28.83 ± 11.26		
>1500	166 (34.16)	26.37 ± 10.74		
Personal income (RMB/mo)				
<2000	149 (30.66)	27.81 ± 9.37	0.850	.428
2000∼3000	122 (25.10)	27.85 ± 10.12		
>3000	215 (44.24)	29.01 ± 10.19		
Frequency of children's visits				
Living with their children	154 (31.69)	29.51 ± 9.46	4.374	.005
More once a week	132 (27.16)	30.14 ± 11.53		
Once to 3 times a year	164 (33.74)	26.36 ± 11.19		
Less once a year	36 (7.41)	25.91 ± 9.79		
Take care of grandchildren				
Yes	148 (30.45)	31.75 ± 9.78	4.949	.000
No	338 (69.55)	26.86 ± 10.13		
Number of hobbies				
0	186 (38.27)	27.03 ± 9.44	5.909	.003
1∼2	206 (42.39)	28.21 ± 9.78		
≥3	94 (19.34)	31.26 ± 10.29		
Group activity frequency				
Regular participation	176 (36.21)	31.02 ± 11.21	10.042	.000
Occasional participation	198 (40.74)	27.49 ± 9.92		
Nonparticipation	112 (23.05)	25.67 ± 10.25		
Effect of epidemic on medical treatment				
No effect	316 (65.02)	29.01 ± 10.52	1.821	.163
A certain extent affect	138 (28.40)	27.14 ± 9.83		
Deep effect	32 (6.58)	27.06 ± 11.36		
Access to medical care				
Online consultation	132 (27.16)	28.62 ± 9.59	0.974	.378
Out-patient medical treatment	265 (54.53)	27.84 ± 9.87		
Treatment for oneself by purchasing medicine	89 (18.31)	29.47 ± 10.33		
Support level for pandemic prevention and control				
Nonsupport	0 (0.00)	–	−0.225	.798
General support	121 (24.90)	28.17 ± 9.78		
Strongly support	365 (75.10)	28.42 ± 9.19		

MUNSH = Memorial University of Newfoundland Happiness.

### 3.3 Multivariate analysis of MUNSH scores of the elderly patients with chronic diseases in Beijing

The multiple linear regression analysis was conducted for MUNSH scores of elderly chronic diseases patients (proceed with *α*_in_ = 0.05, *α*_out_ = 0.10) and the assigned values of each variable, as shown in Table 2. The multiple linear regression indicated that the education level, number of chronic diseases, medical expenses, frequency of children's visits, taking care of grandchildren or not and group activity frequency significantly affected the well-being of patients with chronic diseases during COVID-19 postpandemic period in Beijing (*P* < .05), as shown in Table 3.

**Table 2:** Independent variable assignment.

Variable	Assignment
Age	Enter the actual value
Education level	Primary school or below = 1, junior and senior high school = 2, university or college education = 3
Number of chronic diseases	Enter the actual value
Medical time	Enter the actual value
Medical expenses	<300 RMB/mo = 1, 300∼1500 RMB/mo = 2, >1500 RMB/mo = 3
Frequency of children's visits	More once a week = 1, Once to 3 times a year = 2, Less once a year = 3
Take care of grandchildren	No = 1, Yes = 2
Number of hobbies	Enter the actual value
Group activity frequency	Nonparticipation = 1, occasional participation = 2, regular participation = 3

**Table 3:** Multivariate analysis of MUNSH scores of the elderly chronic disease patients.

Variable	Partial regression coefficient	*SE*	*Beta*	*T*-value	*P*-value
Constant	25.523	2.362		10.806	<.001
Age	0.062	0.019	0.023	2.695	.084
Education level					
Primary school or below (control group)					
Junior and senior high school	0.873	0.174	0.189	4.619	.124
University or college education	2.127	0.339	0.453	6.274	.013
Number of chronic diseases	−0.479	0.105	−0.258	4.562	.036
Medical time	−0.367	0.091	−0.213	4.033	.074
Medical expenses					
<300 RMB/mo (control group)					
300∼1500 RMB/mo	−0.863	0.224	−0.372	3.853	.106
>1500 RMB/mo	−2.232	0.351	−0.583	6.359	<.001
Frequency of children's visits					
More once a week (control group)					
Once to 3 times a year	−2.966	0.623	−0.348	4.761	.028
Less once a year	−3.527	0.501	−0.725	7.040	<.001
Take care of grandchildren	3.582	1.036	0.579	3.458	<.001
Number of hobbies	0.521	0.237	0.318	2.918	.092
Group activity frequency					
Nonparticipation (control group)					
Occasional participation	2.196	0.342	0.394	6.421	.043
Regular participation	3.784	0.298	0.617	12.678	<.001

*R* = 0.457, *R* ^*2*^ = 0.206, adjust the *R* ^*2*^ = 0.192, *F* = 32.439, *P* < .05. MUNSH = Memorial University of Newfoundland Happiness.

## 4 Discussion

Global research data indicated that all types of populations have no immunity of novel coronavirus especially the elderly who accompanied with underlying medical conditions including asthma, diabetes, high blood pressure, respiratory disease and heart disease had higher risk of infection of novel coronavirus.^[8]^ China as one of the most populous countries has a large aging population base while the elderly population above 60 years old have reached 250 million by 2018, which has brought great challenges to workers of prevention and treatment of chronic diseases during the COVID-19 pandemic. Therefore, in the early stage of the outbreak, the elderly especially those with the above-mentioned chronic diseases, even the disease prevention and control personnel will inevitably face huge physical and mental pressure.^[9]^ The domestic pandemic was at a stage of preventing imported case in the postpandemic period and daily life was gradually returning to normal by October 2020 since Chinese government took decisive measures of joint prevention and control. However, many countries abroad are still in a period of increasing confirmed cases, so we still have to remain vigilant against the epidemic.

The well-being of the elderly as an important indicator reflects the quality of life about the elderly, which directly affects the value of their life. Relevant study have shown that the higher the happiness of the elderly, the easier it is to integrate into social life and maintain physical health.^[10]^ This study shows that most elderly patients with chronic diseases during COVID-19 postpandemic period in Beijing were at a high well-being level, while only 11.93% of them have low well-being levels, suggesting that the quality of life of most elderly patients with chronic diseases get cogently guaranteed as a result of potent measures and a superior social system in China.^[11]^ The factors influencing well-being levels of elderly patients with chronic diseases during COVID-19 postpandemic period, research revealed it was bound up with education level, number of chronic diseases, medical expenses, frequency of children's visits, whether to take grandchildren or not and group activity frequency, suggesting the well-being level and quality of life of elderly patients with chronic diseases required for enhancing as provided research foundation for taking corresponding measures as well.^[12]^

Our investigation discovered that subjective well-being level of chronic disease elderly was related to degree of education, elderly who accepted university or college education generally had a high subjective well-being level. Most of the elderly lack the knowledge of COVID-19, however the highly educated elderly have the ability to accept fresh things and adapt to the change of external environment quickly resulting in keeping optimistic and right attitude.^[13]^ Since the novel coronavirus is susceptible to the elderly subsequently the condition turns to be severe after infection, especially for the elderly patients with chronic diseases, to some extent, it aggravates their psychological stress, and may produce anxiety, depression and other negative emotions.^[14]^ Whereas old people with higher education could update current information through reading books or newspapers, utilizing the network and social media thus releasing negative emotions to maintain their high level of happiness.^[15]^

The number of chronic disease as a significant factor affecting the well-being level of elderly patients with chronic diseases had been proved by a study based on the well-being level of elderly chronic patients in Changsha city.^[16]^ With the improvement of living standards, people's diet eating habits and lifestyle have changed along with the incidence of chronic diseases rising in China, particularly the elderly are the high-risk group of chronic diseases.^[17]^ Most chronic diseases are life-long diseases, for elderly patients the suffering caused by long-term illness is easy to rise anxiety, depression and other bad emotions due to their relatively lengthy course of disease and accompanied by a variety of comorbidities.^[18]^ The survey found that elderly patients with chronic diseases as the novel coronavirus susceptible group, the number of chronic diseases was negatively correlated with the well-being level of elderly patients with chronic diseases performing as the higher number of chronic diseases in the elderly, the lower their well-being level, it indicates that we were required to pay more attention to the changes in the condition and daily protection of elderly chronic patients during postpandemic period.

This survey discovered that medical expenses were negatively correlated with the well-being level of elderly chronic patients meaning the higher the medical expenses, the lower the happiness level will be. Research data domestic and overseas manifested that medical costs have become one of the most important factors of well-being level of elderly chronic patients.^[19]^ The major economic income of elderly in China come from pension and their adult children, the higher economic income means abundant medical security and lower psychological stress for chronic disease elderly.^[20]^ During the COVID-19 period, the elderly had to spend a certain amount expense to cure disease and maintain their physical health while suffering and limited activity disturbed their life. Without sufficient financial resources to support the medical costs of chronic diseases can bring financial burden for individuals and families, as reducing the life satisfaction of elderly patients accompanied by a forceful negative psychological impact.

In the postpandemic period, the well-being level of elderly chronic patients is tightly related to the frequency of children's visits and having grandchildren, suggesting that the care of children and their descendants is particularly important for the mental health and life attitude of elderly chronic patients.^[21]^ Studies show that frequency of children's visits as less 3 times per year was negatively correlated with the well-being level of the elderly, children's visits and caring can greatly relieve the negative emotions of the elderly and then obtain more psychological comfort and support. In the meantime, the well-being level of chronic disease elderly with grandchildren significantly increased. For the elderly, skip-generation raising promotes their behavioral activities to improve their physical health. In addition, the interaction with grandchildren can the well-being level by improving the life satisfaction of the elderly in terms of psychological aspect.^[22]^

We explored that the frequencies of elderly group activity were positively correlated with their subjective well-being level presenting as the elderly chronic patients who participated in group activities regularly had higher well-being level. Social activities had an energetic impact for elderly life and interpersonal relationships was closely related to life satisfaction during COVID-19 postpandemic period. The elderly chronic disease patients got emotional support to enrich their spiritual and cultural life through participating in group activities consequently relieved the negative emotions caused by physical discomfort and pandemic influence.^[23,24]^

The strengths of this study. First, the samples we selected have preferable representativeness. The research methods adopted in this study is a cross-sectional study, and the samples we investigated were from the population, which was a good representation. Second, the research results remind us that some easily overlooked factors such as education level, the number of chronic diseases, medical expenses, the frequency of children's visits, whether to take care of grandchildren and the frequency of group activities may be key points that affect the mental health of the elderly with chronic diseases. Therefore, the results of our study have strong promotion significance, which is also helpful for government to formulate relevant policies about elderly chronic patients. However, it should be noted that our research also has certain limitations. In its current form, there is a huge population bias, and the results may not be generalizable. First, the sample size is relatively small and due to the limitations of cross-sectional studies, we can only investigate elderly patients chronic in some areas of Beijing. Second, this study only investigated the well-being level of elderly chronic patients, as well as the well-being level of other normal healthy people and patients with other types of diseases are also worthy of attention. Last, it lacked in-depth research on intervention methods for elderly patients with low well-being. In today's society with advanced science and technology, we can use a variety of media to promote the conclusion, such as the Internet, books, newspapers, TV and news. Under the continuing situation of the pandemic, we can call for more people to pay attention to the mental health and well-being level of elderly patients with chronic diseases, which not only can make up for the population bias, but also can reflect the significance.

In conclusion, most elderly patients with chronic diseases had moderate or above sense of well-being during COVID-19 postpandemic period, but we should still pay attention to the mental health of these patients, especially those elderly chronic patients with low education level, much comorbidity, medical expenses >1500 RMB/mo, less visits by children, not take care of grandchildren and never participate in group activities. And we should still pay more attention to the physical and the mental health of elderly patients with chronic disease.

## Author contributions

**Conceptualization:** Yang Jiao, Chen Wu, Yu-Xuan Liu, Tie-Jun Liu.

**Funding acquisition:** Yang Jiao, Xu-Ling Yan, Tian Zhou.

**Investigation:** Yu-Xuan Liu, Yu-Xi Zhao, Lan-Ying Sun, Jia-Ning Xi.

**Methodology:** Yu-Xuan Liu, Ping Rao, Tian Zhou.

**Project administration:** Lan-Ying Sun, Yang Jiao, Chen Wu.

**Resources:** Yang Jiao, Ping Rao, Tie-Jun Liu.

**Supervision:** Hong Zeng, Xu-Ling Yan.

**Validation:** Yang Jiao, Jia-Ning Xi.

**Visualization:** Yang Jiao, Tian Zhou, Yang-Yang Gao.

**Writing – original draft:** Chen Wu, Yu-Xuan Liu, Tie-Jun Liu.

**Writing – review & editing:** Yu-Xuan Liu, Lan-Ying Sun, Xu-Ling Yan.
